# Optical Analog to Electromagnetically Induced Transparency in Cascaded Ring-Resonator Systems

**DOI:** 10.3390/s16081165

**Published:** 2016-07-25

**Authors:** Yonghua Wang, Hua Zheng, Chenyang Xue, Wendong Zhang

**Affiliations:** Key Laboratory of Instrumentation Science & Dynamic Measurement, Ministry of Education, North University of China, Taiyuan 030051, China; huazaisss@foxmail.com (Y.W.); 18334792568@163.com (H.Z.); wdzhang@nuc.edu.cn (W.Z.)

**Keywords:** coupled resonator induced transparency, electromagnetically induced transparency, ring resornator, silicon waveguide

## Abstract

The analogue of electromagnetically induced transparency in optical methods has shown great potential in slow light and sensing applications. Here, we experimentally demonstrated a coupled resonator induced transparency system with three cascaded ring coupled resonators in a silicon chip. The structure was modeled by using the transfer matrix method. Influences of various parameters including coupling ratio of couplers, waveguide loss and additional loss of couplers on transmission characteristic and group index have been investigated theoretically and numerically in detail. The transmission character of the system was measured by the vertical grating coupling method. The enhanced quality factor reached 1.22 × 10^5^. In addition, we further test the temperature performance of the device. The results provide a new method for the manipulation of light in highly integrated optical circuits and sensing applications.

## 1. Introduction

The electromagnetically induced transparency (EIT) effect is a nonlinear effect which occurs in the interaction process between light and material [1]. Due to strong material dispersion caused by quantum interference, a transparency peak will emerge in the absorption curve and the group velocity of light can be rapidly slowed down [2,3,4] and therefore the EIT effect has shown great potential in various promising applications, including slow light control, quantum information processing and sensing technology [5,6]. However, the realization of EIT in atomic systems is a tough task since some restrictions must be strictly fulfilled. Recently, researchers have found that the EIT-like spectrum can also be generated in some other structures which are easier to realize, such as optical resonators [7,8,9] and Fano metamaterials [10,11,12,13,14,15]. For the case of EIT, the transparency window is caused by the reduced absorption, due to the quantum destructive interference between the transitions from the two dressed states, into a common energy level. Similarly, the EIT-like effect generated by optical resonators works by means of coherent interference between the resonating modes which produces optical transparency inside the absorption window. Compared to the EIT in atomic systems, the analogue of electromagnetically induced transparency with optical resonators has many remarkable advantages such as simpler structure, smaller device size and being easier to design, which mean it has enormous potential for applications in optical sensors [16,17] and optical delay lines [18,19]. The optical EIT-like effect has been experimentally realized in many optical structures including coupled fiber systems [20], coupled fused-silica spheres [21] and optical parameter oscillator cavities [22]. In these structures, the linewidths of the induced window can be extremely narrow. The drawback of these devices is the bulky and incompact structure which is hardly present for integrated applications. The on-chip integrated all-optical analogue of EIT effect has been realized in photonic crystals [23,24] and silicon microrings [8,25]. However, due to the small size of these structures, it is challenging to detune optical cavity for controlling of the resonant interaction between the two optical pathways. For instance, the two paralleled resonators [26] require an ~8 nm perimeter difference between the two rings, which is normally very challenging to control in the fabrication.

Here, we theoretically and experimentally demonstrate an all-optical analog to EIT on a chip in a three-cascaded-ring system. The mathematical model was established by using transfer matrix method. The bandwidth of the central transmission peak and maximum group index was analyzed with various parameters. By optimizing the coupling angular deviation and the coupling strength of the three resonators, a significant transparency window has been experimentally obtained. The effect of the coupling strength between the resonators has been demonstrated, indicating that the EIT-like window has a good sensitivity to the temperature and stress. The experimentally measured quality factor of the transparency system has reached 1.22 × 10^5^, which is much larger than the Q factor in single resonators and in the two-paralleled-ring EIT-like systems. In this three-cascaded-ring system, the EIT-like spectrum is much easier to achieve since we can change the coupling angular deviation and the coupling distance to obtain a good coherent interference between the resonators, which can make up for the deficiency in the fabrication. Compared to the parallel double-ring coupled resonator, a narrower transmission spectrum and higher group index has been achieved in our system, which means a better sensitivity and a slower light velocity for sensing applications.

## 2. Structural Design

**Figure 1:**
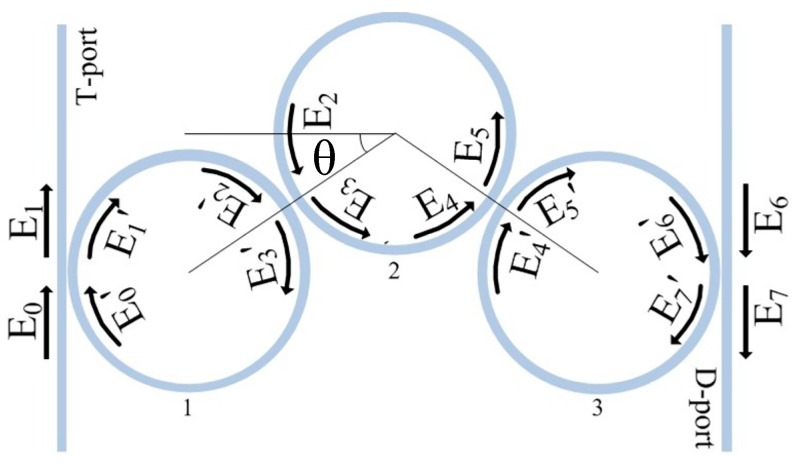
Schematic of three-ring coupled resonator.

Figure 1 shows the schematic structure of three-cascaded-ring system which consists of two straight waveguides and three microrings. The radius of microrings is 20 μm. E_0_ is the input optical field, E_1_ is the output of the through port and E_7_ is the output of the drop port.

The transmission matrix of this structure could be written as:

(1) $$\left[\begin{array}{c} E_{6}\\ E_{7} \end{array}\right]=P_{4}Q_{3}P_{3}Q_{2}P_{2}Q_{1}P_{1}\left[\begin{array}{c} E_{0}\\ E_{1} \end{array}\right]=\left[\begin{array}{cc} A_{11} & A_{12}\\ A_{21} & A_{22} \end{array}\right]\left[\begin{array}{c} E_{0}\\ E_{1} \end{array}\right]$$

where $P_{i}=\frac{1}{i\sqrt{k_{i}}}\left[\begin{array}{cc} -\sqrt{1-k_{i}} & 1/\sqrt{1-r_{i}}\\ -\sqrt{1-r_{i}} & 1/\sqrt{1-k_{i}} \end{array}\right]$, *k_i_* and *r_i_* are the coupling coefficient and insert loss of coupler *i* respectively. $Q_{1}=Q_{3}=\left[\begin{array}{cc} 0 & e^{-i\theta_{1}R\beta }\\ e^{i\theta_{2}R\beta } & 0 \end{array}\right]$ is the transmission matrix of ring1 and ring3. $Q_{2}=\left[\begin{array}{cc} 0 & e^{-i\theta_{3}R\beta }\\ e^{i\theta_{4}R\beta } & 0 \end{array}\right]$ is the transmission matrix of ring2. *θ_i_* is the phase shift of the *i-*th ring, $\beta =n\frac{\omega }{c}-ia$ is propagation constant, *n* is effective refractive index, ω is the angular frequency of light, c is speed of light, a is loss of waveguide. The transfer function of through port and drop port can be expressed as:

(2) $$T_{through}=-\frac{E_{1}}{E_{0}}=-\frac{A_{11}}{A_{12}}$$

(3) $$T_{drop}=\frac{E_{7}}{E_{0}}=A_{21}-\frac{A_{11}A_{22}}{A_{12}}$$

**Figure 2:**
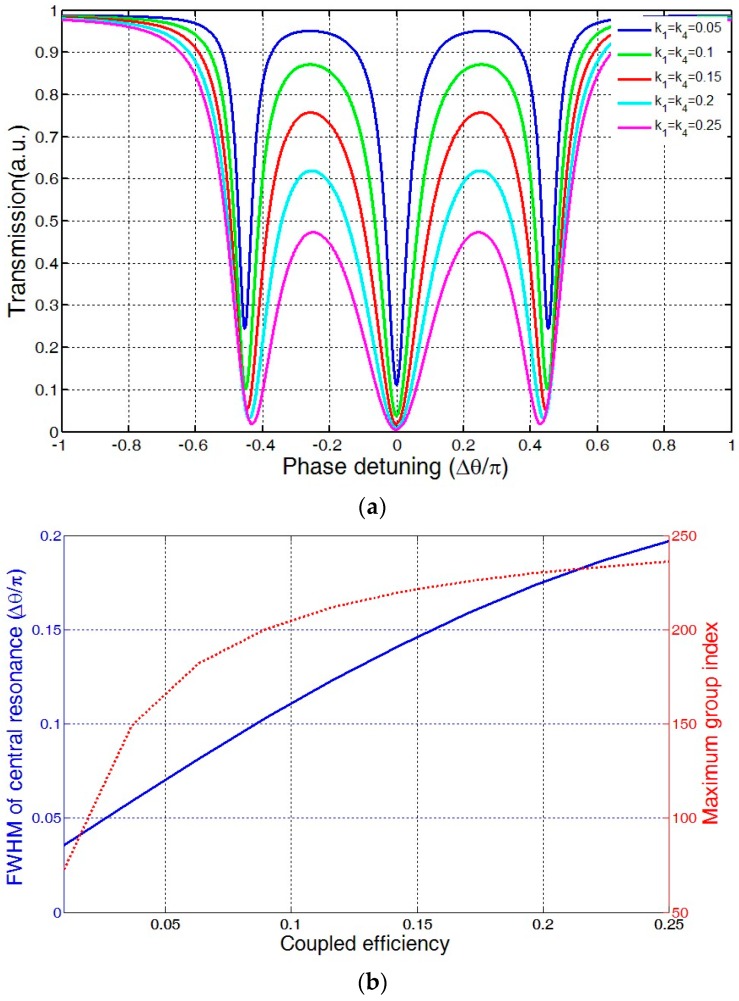
Effect of waveguide-microring coupling coefficient: (**a**) on transmission spectrum (**b**) on FWHM (full width at half maximum) of central resonance and maximum group index.

Figure 2a illustrates the power transmission of three-ring coupled resonator with k_1_ = k_4_ = 0.05, 0.1, 0.15, 0.2, 0.25. It can be seen that there would be three absorption bands in the transmission. As the coupling coefficient increases, resonant depth would evidently decrease. At k_1_ = k_4_ = 0.25, the maximum power transmittance of central resonance has diminished to 0.46. Figure 2b shows the effect of waveguide-microring coupling coefficient on the FWHM (full width at half maximum) of the central resonance and maximum group index. With an increase of the coupling coefficient, both FWHM and maximum group index would increase.

**Figure 3:**
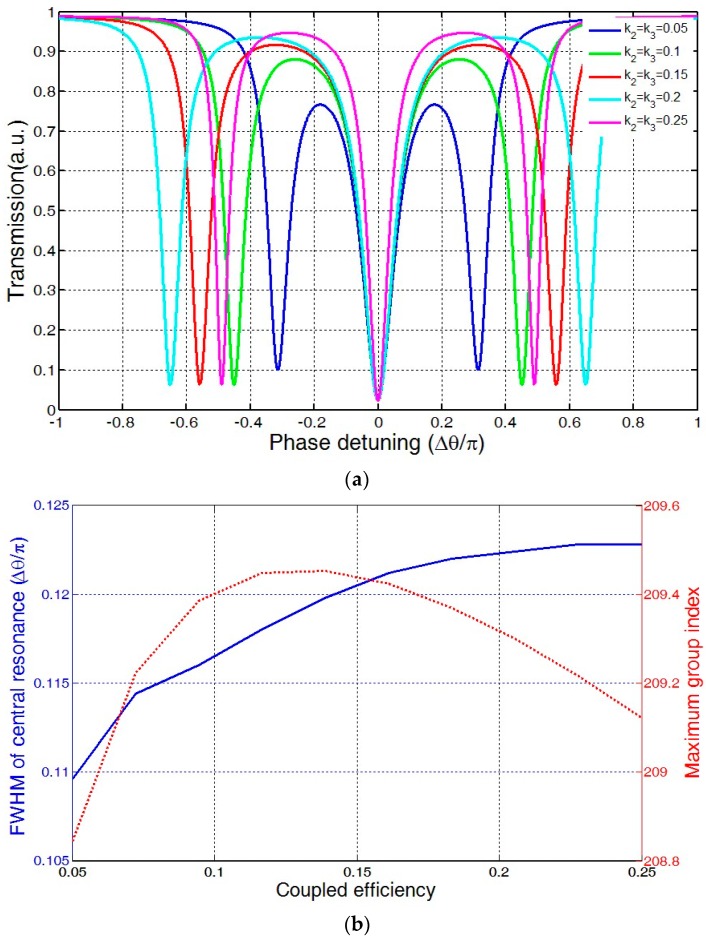
Effect of ring-ring coupling coefficient: (**a**) on transmission spectrum (**b**) on FWHM of central resonance and maximum group index.

The relation between microring-microring coupling coefficient and transmission spectrum is shown Figure 3a. It can be seen that with the increase of k_2_ = k_3_, the position of central resonance is almost unchanged, but the two side-resonances move away from central resonance gradually. Figure 3b shows the effect of microring-microring coupling coefficient on FWHM of central resonant and maximum group index. After increasing of k_2_ = k_3_, the FWHM of central resonance was quickly extended while the maximum group index was raised at first and then declined.

**Figure 4:**
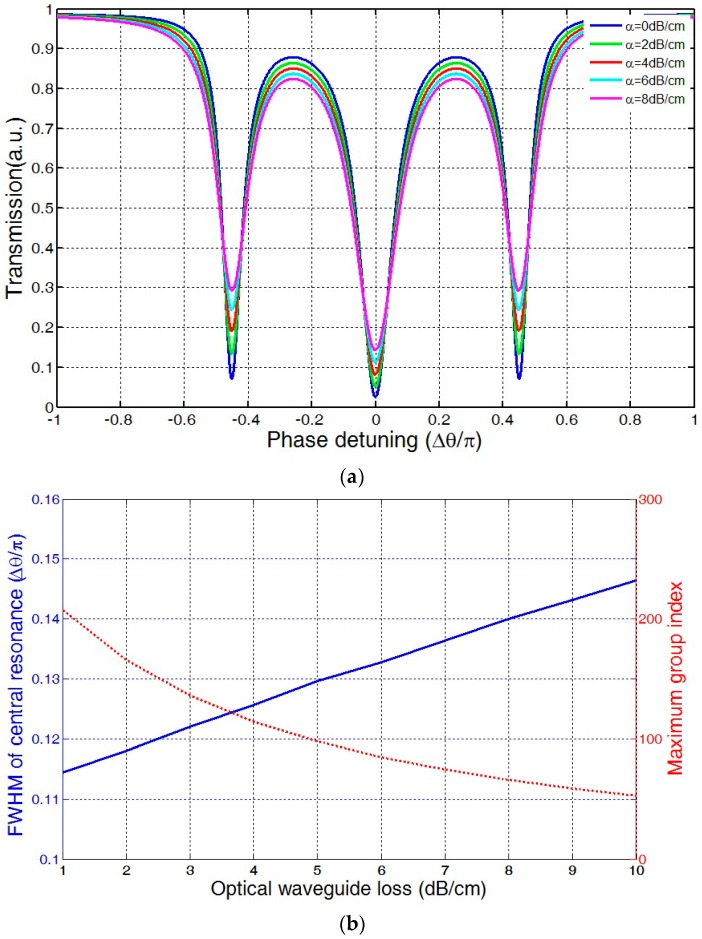
Effect of waveguide loss: (**a**) on transmission spectrum (**b**) on FWHM of central resonance and maximum group index.

Figure 4a shows the transmission of the three-ring coupled resonator with different waveguide losses. It can be clearly seen that, as the waveguide loss increases, the resonant depth of the three resonances would decrease. Figure 4b shows the effect of the optical waveguide loss on FWHM of the central resonance and maximum group index. With the increase of optical waveguide loss, the FWHM of the central resonance would increase, and, in contrast, the maximum group index would decrease.

## 3. Device Fabrication and the Experiment

**Figure 5:**
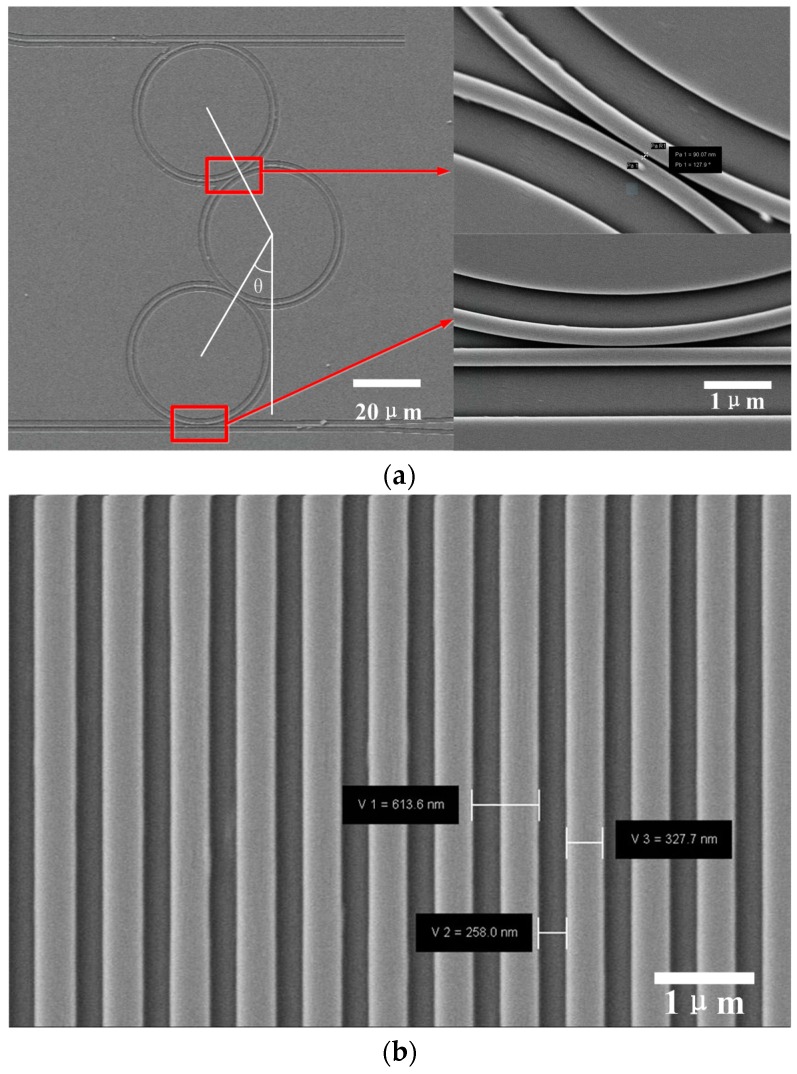
SEM of the cascaded rings and the gratings: (**a**) rings and straight waveguide (**b**) the grating couplers.

The scanning electron microscopy (SEM) graphs of three-ring coupled resonators are shown in Figure 5. The device was fabricated on commercial SOI wafers, having a silicon thickness of 220 nm and a buried-oxide thickness of 3 μm. Electron beam lithography (JBX5500ZA) and induction coupler plasma etching process (STS HRM ICP) were used to fabricate the three-ring coupled resonator. The bus waveguide and the cascaded ring resonators were fully etched, with a depth of 220 nm and a width of 450 nm, to guide single-mode transverse electric (TE) light. The radii of the two ring resonators were designed to be 20 μm. At both ends of the bus waveguide, a pair of grating couplers was fabricated with a 600 nm period, in order to couple light in and out of the devices. The distance between the straight waveguide and the microring was 110 nm, which is the optimum distance as proven from past experience. The gap between the microring and microring was designed from 70 nm to 200 nm. The coupling angular deviation θ was designed from 0° to 60°. To decrease the loss of the waveguide, 300 °C to 1200 °C thermal oxidation procedure was used. Subsequently, the Buffered Oxide Etch and 1000 °C annealing with nitrogen were used to obtain a smoother waveguide surface, which means a higher Q factor. The enlarged views of Figure 5a show that the straight waveguides and the rings have good surface roughness.

**Figure 6:**
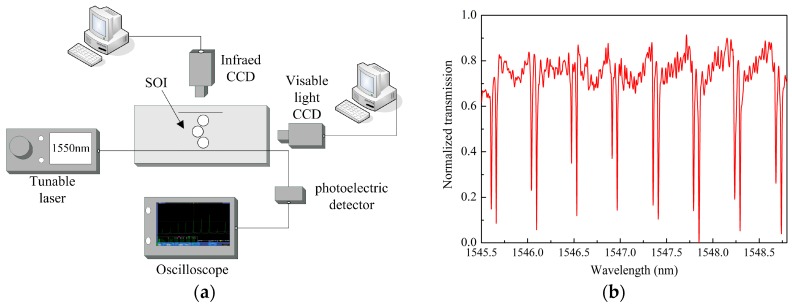
(**a**) Device measurement setup and (**b**) scanning curve of the structures with θ = 30°.

The performance of the device was tested by vertical grating coupling method. The experimental system is illustrated in Figure 6a. The generation of the transparency curve is obtained by sweeping the frequency of the tunable laser (New Focus TLB-6728-P, Irvine, CA, USA, linewidth is less than 200 kHz) with the resolution of 0.01 nm. Light export from the tunable laser source is polarization-tuned by a polarization controller and injected into the grating coupler using a 75° lensed fiber and aligning by a precision translation stage (New Focus M-461-XYZ-M). The output light is detected by a photoelectric detector (New Focus 1811) and displayed on the digital oscilloscope (Tektronix MDO3054, Beaverton, OR, USA). Figure 6b shows the transmission spectrum from the through port of the structure with θ = 30° and the gap between the resonators of 110 nm.

**Figure 7:**
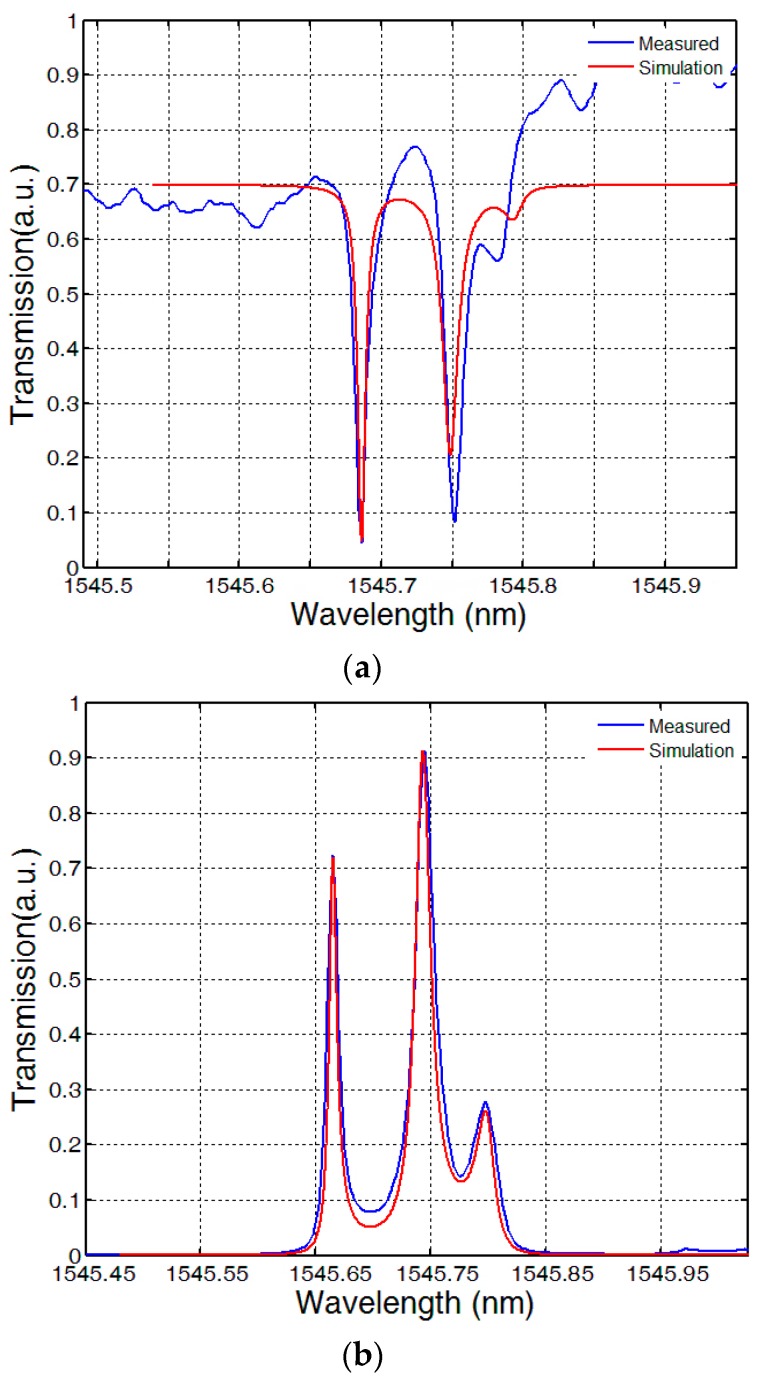
The measured transmission spectrum of (**a**) the through port and (**b**) the drop port. The **blue** line is the experimental result curve and **red** line is theoretical fitting curve.

In the theoretical analysis, the coupling angular deviation θ has no influence on the transparency window, but in the actual fabrication it can change the destructive interferences between the responses of the three rings due to the scattering loss and the bending loss of the waveguide. Utilizing this character, we have fabricated different coupling angular deviation and obtained a significant transparency peak with the θ = 30°. One of the transparency windows in the wavelength axis with the theoretical fitting curves is shown in Figure 7. It can be observed that the transmission peak splits into three narrow peak in both the through port and the drop port. From the theoretical analysis we observe that the transmission character of our system is extremely sensitive to the radius of the microring. Due to machined error, there would be slight differences between the radius of the microrings which would lead to the asymmetry of the two side-resonance. The FWHM of the central resonance is as narrow as 0.0125 nm, corresponding to a quality factor of 1.22 × 10^5^. The red line in Figure 7 is the theoretical fitting with k_1_ = k_4_ = 0.15, k_2_ = 0.108, k_3_ = 0.15, r_1_ = 20 μm, r_2_ = 19.997 μm and r_3_ = 20.0072 μm, which shows good agreement with the experimental result.

**Figure 8:**
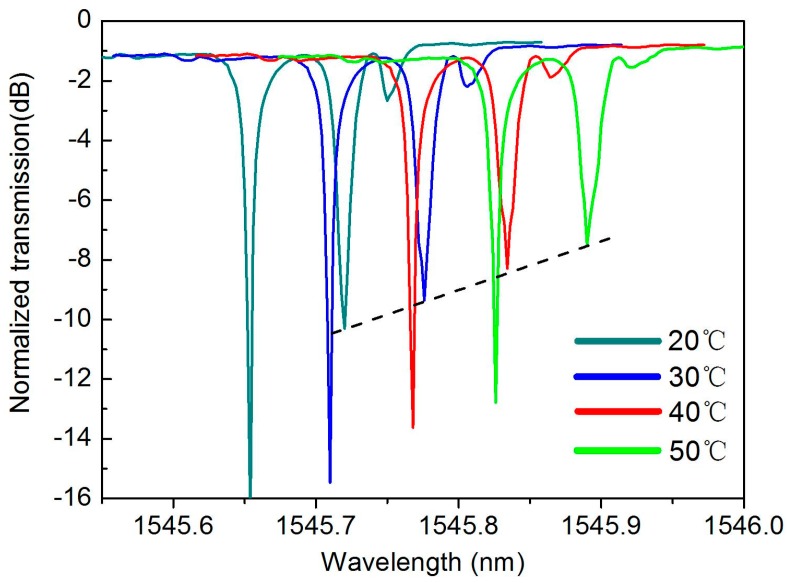
The response of transparency window in different temperatures.

Since the EIT-like window in our device has a significant sensitivity to the change of the ring perimeter, we are convinced that the EIT-like system can be used as a temperature sensor, in which the resonant strength of the transparency window will be changed by the thermal expansion effect. Therefore, we have further tested the response of transparency window in different temperatures, as shown in Figure 8.

The temperature sensors that use the single resonator have been reported [26,27], in which the temperature variation was measured by monitoring the shift in the resonant wavelength of the silicon resonator. However, the temperature variations will induce the thermo-optic effect and the thermal expansion effect simultaneously. This means the shift in the resonant wavelength will be influenced by both the changed effective index and the changed ring perimeter. Since we cannot verify the actual effective index and the group index in the fabricated silicon ring resonators, it is not accurate if we only consider the shift in the resonant wavelength. While in our scheme, the temperature not only changes the resonant shift, but also changes the resonance strength. Therefore, utilizing our EIT-like scheme for temperature sensing is a promising method.

## 4. Conclusions

In summary, EIT-like effect has been demonstrated theoretically and experimentally in the cascaded three-ring coupled resonators on a silicon chip. The influence of parameters including coupling coefficient, waveguide loss, addition loss of couplers on EIT-like effect have been studied. EIT-like resonance is experimentally observed in the cascaded three-ring coupled resonator with a quality factor of 1.22 × 10^5^. The experimental results agrees with the theoretical analysis well. The temperature performance of the device is further studied. This structure is promising for applications in integrated optical sensors and optical delay lines.
